# Mentalising skills in generic mental healthcare settings: can we make our day-to-day interactions more therapeutic?

**DOI:** 10.1192/bjb.2017.29

**Published:** 2018-06

**Authors:** H. J. Welstead, J. Patrick, T. C. Russ, G. Cooney, C. M. Mulvenna, C. Maclean, A. Polnay

**Affiliations:** 1 Lansdowne Psychotherapy Service, Glasgow, UK; 2 State Hospital, Carstairs, UK; 3 Royal Edinburgh Hospital, Edinburgh, UK; 4 Gartnavel Royal Hospital, Glasgow, UK; 5 University of Glasgow, Glasgow, UK

## Abstract

**Aims and method:**

Caring for patients with personality disorder is one of the biggest challenges in psychiatric work. We investigated whether mentalisation-based treatment skills (MBT-S) teaching improves clinicians' understanding of mentalising and attitudes towards personality disorder. Self-report questionnaires (Knowledge and Application of MBT (KAMQ) and Attitudes to Personality Disorder (APDQ)) were completed at baseline and after a 2-day MBT-S workshop.

**Results:**

Ninety-two healthcare professionals completed questionnaires before and after training. The mean within-participant increase in scores from baseline to end-of-programme was 11.6 points (95% CI 10.0–13.3) for the KAMQ and 4.0 points (1.8–6.2) for the APDQ.

**Clinical implications:**

MBT-S is a short intervention that is effective in improving clinicians' knowledge of personality disorder and mentalisation. That attitudes to personality disorder improved overall is encouraging in relation to the possibility of deeper learning in staff and, ultimately, improved care for patients with personality disorder.

**Declaration of interest:**

None.

Personality disorder is of major clinical importance: a third of psychiatric out-patients and over half of in-patients are estimated to fulfil the criteria for personality disorder.^1^ Borderline personality disorder (BPD) is the most prevalent personality disorder in the non-forensic setting^2^^,^^3^ and is associated with intensive use of psychiatric services and frequent admissions.^4^^,^^5^ Caring for patients with BPD can be experienced as emotionally challenging,^6^^,^^7^ and these patients are perceived by some clinicians to be manipulative, attention-seeking or threatening,^8^ making it hard to maintain a therapeutic stance towards them. In turn, patients may experience staff as prejudicial and unhelpful.^1^ Negative staff attitudes towards personality disorder are associated with poorer therapeutic relationships, reduced standards of care and overall poorer outcomes.^7^^,^^9^

## Mentalisation-based treatment

A central component of BPD is that of a hypersensitive attachment system.^10^ At times of stress, patients will more readily seek proximity with a caregiver. Yet the behaviours that sometimes accompany this proximity-seeking, such as self-harm or suicidal acts, can seem irrational and frustrating to the caregiver, who might then lose their own ability to mentalise, for example, through the development of a judgemental attitude towards the patient or a belief that they are undeserving of care.

## Mentalising in the context of generic mental health services

A mentalising stance involves having an inquisitive, empathic, open-minded and ‘not-knowing’ approach to mental states, and an ability to consider alternative perspectives. Mentalisation-based treatment (MBT) provides a framework to help healthcare staff understand their attitudes and feelings, and teaches ways to restore mentalising in both the professional and the patient. Bateman suggests that effective MBT skills can be gained through limited additional training and with moderate levels of supervision.^11^

## Teaching mentalising skills

There has been a recent governmental drive to improve systems for staff support and supervision in managing these patients, and to develop courses teaching staff to better address patients' needs.^1^^,^^7^^,^^12^ In that spirit, a 2015 pilot study found that a brief (4 h) teaching intervention in MBT skills improved psychiatry core trainees' understanding of mentalising and their attitudes to personality disorder.^13^ To test whether this finding is replicated in a larger sample and whether it generalises to other professional groups, we conducted a before-and-after comparison of mental healthcare staff who underwent a 2-day course in MBT skills (MBT-S). To our knowledge, this is the first published quantitative evaluation of MBT-S. It addresses an important clinical question: alongside MBT constituting a specialist treatment, does a mentalising skills intervention provide an accessible theoretical framework for staff working in generic mental health settings?

## Method

### Participants

Participants in the MBT-S courses included doctors, nurses, psychologists and allied healthcare professionals working in various mental health services within NHS Lothian. Once a clinical team was identified by the course leaders as potentially able to benefit from the course, all staff members working in that service were invited to take part in the training. In some cases, individuals and teams self-selected to attend the training.

All participants attending the first day of training were eligible to be included in the study. Participants who only attended the second day were excluded.

### Intervention

MBT-S is aimed at generic mental health practitioners and is taught in an accessible format to help staff in difficult day-to-day interactions, with the aim of fostering more effective therapeutic relationships with their patients. The teaching is based on a MBT skills package developed by the Anna Freud Centre^14^ in conjunction with MBT Scotland. It was adapted by two of the authors (J.P. and C.M.), and these adaptations were authorised by the Anna Freud Centre.

The MBT-S training was delivered on two single days separated by a few weeks to allow participants to practise their skills and complete allotted tasks. The format was a combination of didactic teaching, role-play and DVD clips. It included a theoretical framework that explains attachment theory and how personality disorder and mentalising difficulties develop, as well as specific MBT techniques designed to strengthen both the patient's and the professional's ability to mentalise in stressful situations. The training was delivered in the Psychotherapy department at the Royal Edinburgh Hospital by Anna Freud Centre-accredited trainers, including two of the authors (J.P. and C.M.).

### Aims

This study aimed to answer the following questions.

(a) Is a 2-day course in MBT-S effective in improving general mental health practitioners' understanding of mentalising?
(b) What effect, if any, does it have on their attitudes to personality disorder?
(c) How do different professional groups compare in terms of outcomes?

### Outcomes

Anonymised self-report questionnaires were given to participants by the study authors immediately prior to commencing the programme and again directly after the programme ended. Data on the participants' professional groups were collected from five of the six training courses.

The main outcome measure was the Knowledge and Application of MBT Questionnaire (KAMQ; see Appendix 1) (A. Williams, C Cahill, J Patrick, personal communication, 2015). This 20-item questionnaire asks about knowledge of MBT (e.g. ‘A key component of mentalising is thinking about people's attachment relationships’) and how to apply MBT techniques, using a five-point Likert scale from ‘strongly disagree’ to ‘strongly agree’. A higher score indicated better knowledge of mentalising concepts and MBT techniques, with a maximum total score of 100 points. Work is currently in progress describing the development of this questionnaire and evaluating its psychometric properties.^15^

The secondary measure was the Attitudes to Personality Disorder Questionnaire (APDQ; see Appendix 2), which measures clinicians' attitudes towards people with personality disorder. This questionnaire has 37 items that ask about the intensity of a person's feelings (e.g. ‘I feel understanding towards people with personality disorder’) using a six-point Likert scale, from ‘never’ to ‘always’. A higher score indicated a more positive attitude, with a maximum score of 222 points. The APDQ has good internal consistency (Cronbach's alpha = 0.94) and test–retest reliability (*r* = 0.71).^16^

### Statistical methods

Data were entered into MS Excel by three authors (H.J.W., G.C. and C.M.M.). They were analysed by author T.C.R. using R for Windows 3.2.3. Linear regression was used to compute within-person change in score from baseline to end-of-programme for the KAMQ and APDQ separately. We used unadjusted models to explore the changes in scores over time and then constructed models adjusting for job category (whether different groups had differences in scores at baseline, i.e. had different intercepts) and an interaction term between job category and time (whether different professional groups were affected more than others over time, i.e. had different slopes). Effect sizes (Cohen's D) were calculated in order to examine the magnitude of difference between pre- and post-scores.

### Missing data

An intention-to-treat analysis was carried out. Missing items at baseline were assumed to be missing at random, and the mean score among all responders for that item was entered. For missing end-of-programme items, baseline values were carried forward.

## Results

Six training courses, with a median 16 participants each (range 12–19), were carried out between June 2014 and March 2016. Across all six courses, a total of 92 participants attended the first day of training and so were eligible for the study. All 92 were enrolled and completed the baseline questionnaires. Two participants attended the second day of training only and were excluded from the study.

Across all baseline questionnaires, 77 individual items (1.46% of the total baseline data) were left blank and imputed (mean) values inserted.

Across all completed end-of-programme questionnaires, 139 individual items (2.65% of the total end-of-programme data) were left blank. Eight end-of-programme questionnaires were not completed as the participant did not attend; this accounted for 8.69% of the total end-of-programme data. All end-of-programme missing data were treated in the same way, with their baseline scores being carried forward.

A *post hoc* power calculation suggested that our sample size of 92 at conventional levels of statistical significance (α = 0.05) would have 80% power to detect a small effect size (0.2).

### Data on professional groups

Data on professional groups were not collected for the first of the courses (June 2014) but were collected for all subsequent courses. The 74 participants whose job title was known were categorised according to professional background. The biggest group was nursing, representing 46 (62%) participants. The second largest group was psychology with 12 participants (16%), and the third largest was medical, with eight participants (11%). Other professional backgrounds included occupational therapist (*N* = 3), art therapist (*N* = 3), social worker (*N* = 1) and recreation assistant (*N* = 1).

For the regression analyses using professional groupings, we compared participants with medical and psychology backgrounds with those with a nursing background, to allow large enough samples for the analyses. The justification for combining these two groups was that there are likely to be parallels in the experience and training of participants with a medical and psychological background in relation to personality disorders. The comparison with participants from a nursing background was thought to be of interest. We excluded the smaller groups.

### Outcomes

#### Knowledge and Application of MBT Questionnaire

The mean KAMQ score at baseline was 74.7 points (s.d. = 7.6). There was a mean within-person increase of 11.6 points (95% CI 10.0–13.3) from baseline to end-of-programme. The effect size was 1.2, which was considered a large effect.

#### Attitudes to Personality Disorder Questionnaire

**Fig. 1:**
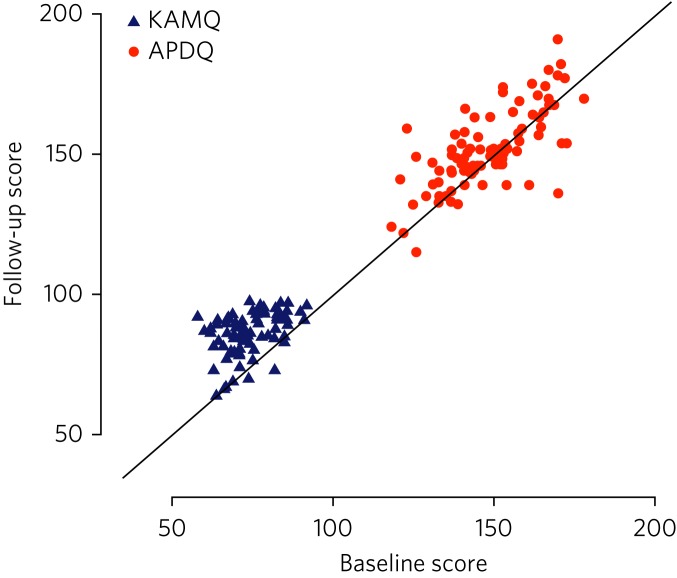
Jacobson plot of within-person change in KAMQ and APDQ scores. Markings above the oblique line indicate an increase in scores from baseline to end of programme.

The mean APDQ score at baseline was 148.7 points (s.d. = 12.3). There was a mean within-person increase in APDQ scores from baseline to end-of-programme of 4.0 points (95% CI 1.8–6.2). The effect size was 0.2, which was considered a small effect. In 23 cases, there was no change in APDQ scores from baseline to end-of-programme; in 42 cases, the scores improved, and in 27 cases, APDQ scores worsened (Fig. 1).

Participants with a medical and psychological professional background had better baseline KAMQ scores when compared jointly with those with a nursing background, but their APDQ scores increased to a lesser degree after the teaching compared with nurses (see Table 1).

**Table 1:** Linear regression model including fixed effects for time and professional group (doctors and psychologists *v.* nurses) and an interaction term between job category and time

KAMQ		APDQ	
Difference in baseline scores: doctors and psychologists *v.* nurses	Difference in score increase from baseline to end-of-programme: doctors and psychologists *v.* nurses	Difference in baseline scores: doctors and psychologists *v.* nurses	Difference in score increase from baseline to end-of-programme: doctors and psychologists *v.* nurses
12.1 (CI 6.0 to 18.4)	−4.1 (CI −8.1 to −0.3)	4.8 (CI −5.1 to 14.8)	−4.8 (CI −9.5 to −0.1)

## Discussion

### Interpretation of results

The main finding of our study is that MBT-S training improved mental healthcare professionals' KAMQ scores to a statistically significant degree and with a large effect size, suggesting that it is an effective way of teaching MBT concepts to staff working in generic mental health settings. Furthermore, this finding is in keeping with recent qualitative research that demonstrated that nursing staff who participated in an MBT-S course felt that it provided a ‘straightforward but empowering skill set’ in working with patients with personality disorder.^17^

Attitudes to personality disorder improved overall to a lesser, albeit still statistically significant, degree. By definition, attitudes are somewhat engrained in someone's personality and professional way of working, so they may, of course, be hard to shift. It is interesting that in 27 cases, the APDQ scores worsened. Although we cannot rule out the possibility that the intervention might have resulted in a genuine worsening of a minority of participants' attitudes towards personality disorder, we think it is plausible that this reflects participants' increased awareness and acceptance (i.e. mentalisation) of negative feelings towards patients (c.f. limitations of the study, below). Alternatively, this may represent an artefact of test–retest reliability.^16^

We note the finding that doctors' and psychologists' APDQ scores improved less than those of nurses. Baseline APDQ scores were higher for doctors and psychologists, so it may be that we are seeing a ‘ceiling effect’ – there is less room to improve from a higher baseline. In the context of previous research,^13^ we think the salient point is that this intervention appeared to be effective for professional groups other than doctors in training.

### Comparison to other literature

The only other study to date to evaluate staff knowledge and application of MBT following MBT skills training was a pilot study that also demonstrated an improvement in KAMQ scores with large effect.^13^

A number of studies have assessed the effect of training on staff attitudes to personality disorder. In a randomised controlled study, Clarke *et al*^18^ compared a psychoeducation programme with an intervention designed to help mental healthcare staff deal with the difficult feelings triggered by working with personality disorder patients (acceptance and commitment training). Participants had responded to an advertisement and volunteered for the free 2-day training. Both forms of training were found to significantly improve APDQ scores immediately post-training compared with baseline (based on their data, we have calculated Cohen's D to be 0.28 and 0.22 respectively), with no statistical difference between the two. The improvements were sustained at 6 months follow-up, although there was a high rate of drop-out resulting in a loss of statistical power.

A study assessing the effects of a 2-h personality disorder awareness workshop on prison staff (*N* = 26) found no significant difference in APDQ scores before and 2 months after the training.^19^

In a systematic review of interventions aimed at improving mental health nurses' skills, attitude and knowledge related to patients with BPD, Dickens *et al*^20^ reviewed eight studies whose interventions ranged from a 90-min lecture to the complete 18-month intensive dialectical behaviour therapy training. None of these studies used the APDQ as an outcome measure. They found that changes in affective outcomes (including attitudes to personality disorder) were usually associated with small effect sizes, although changes in cognitive outcomes (including knowledge) were associated with larger effect sizes.

The outcomes of these studies indicate that these different forms of intervention have generally resulted in small improvements in participants' attitudes and emotional responses to personality disorder, but greater improvements in their knowledge relating to personality disorder. The ability to compare these outcomes with the present paper is limited owing to the use of different methodologies and the absence of studies that directly compare MBT-S with other interventions. Within this limitation, we note that the size of outcomes from the comparative literature mirror the results of the present intervention, MBT-S. This may confer preliminary support for the comparable efficacy of MBT-S.

### Strengths and limitations of the study

To our knowledge, this is the largest quantitative study to evaluate the effects of MBT-S on clinicians. Strengths of the study include the low study drop-out rate (8.7%) and the intention-to-treat analysis.

One possible limitation is linked to the choice of questionnaire. The APDQ has no formal validity data, which limits the interpretation of our results. In addition, the APDQ relies on participants' reporting of feelings: the reporting of positive feelings is linked to a ‘better’ attitude, while the reporting of negative feelings is linked to a ‘worse’ attitude. Yet for clinicians, being aware of negative feelings towards patients is likely to be helpful, as it gives them a chance to consider and reflect on their responses, and makes them less likely to act on feelings in a counter-therapeutic way.^21^ A lower APDQ may not, therefore, indicate a less helpful clinician stance, and *vice versa*. Work is needed to establish benchmarks for the KAMQ – i.e. what constitutes a ‘good’ level of knowledge about mentalising.

That the training was delivered by two of the study's authors introduces the risk of bias. Data entry and analysis were performed by authors who had no role in the delivery or running of the courses, limiting this risk. Some individual participants and mental health teams self-selected to attend the training, which introduces a potential confounding factor. The internal validity would be improved by having a control group. This would pose some practical problems, not least the challenge of providing a convincing 2-day ‘placebo’ training. An alternative would be to have a practice-as-usual control group, who only complete the outcome measures.

There has so far been no longitudinal follow-up of the study's participants. Therefore, we cannot comment on whether the effects of training persist.

### Research and clinical practice implications

Our study suggests that MBT skills teaching is a good way of improving staff knowledge about mentalising skills and is accessible to different professional groups. That attitudes to personality disorder improved overall is also encouraging in relation to the possibility of deeper learning in staff.

Our findings add weight to the need for a larger study of MBT-S that uses both staff and patient outcomes and incorporates a control group. We note the importance of follow-up beyond the intervention to investigate whether effects persist; accordingly, follow-up is planned. Future research should aim to establish the potential influence of teaching mentalising skills on ‘real world’ clinical work, such as better therapeutic relationships or reductions in self-harming behaviour. A recent case study assessing the utility of MBT-informed practice and reflection in the in-patient forensic mental health setting suggested that it may be helpful.^22^

Drawing on guidelines on effective team approaches to working with people with personality disorder,^23^ it would be prudent, in devising an intervention to train a clinical team in MBT skills, to ensure that good principles and structures are in place first. These include making time for formulation,^24^ establishing a structured approach to clinical care (consistency, clarity of staff roles),^25^ and ensuring that good systems of staff support, supervision and reflective practice are in place.^26^

In summary, MBT-S is a short intervention that is effective in improving clinicians' knowledge of personality disorder and mentalisation. Recent UK health policies have urged mainstream mental health services to be more responsive to the needs of individuals with personality disorder.^1^^,^^7^ Our findings suggest that MBT-S might be an effective way to respond to this need, and one that is accessible to a range of professional groups.

## About the authors

**H. J. Welstead** is an ST6 in Psychotherapy at Lansdowne Psychotherapy Service, Glasgow, UK; **J. Patrick**, is a Consultant Psychiatrist in Psychotherapy and Forensic Psychiatry at State Hospital, Carstairs, UK; **T. C. Russ**, is a Consultant Old Age Psychiatrist, Royal Edinburgh Hospital, Co-Director of the Alzheimer Scotland Dementia Research Centre, University of Edinburgh, and an Intermediate Clinical Fellow at the Centre for Dementia Prevention, University of Edinburgh, UK; **G. Cooney** is an ST5 in General Adult Psychiatry at the Kershaw Unit, Gartnavel Royal Hospital, Glasgow, UK; **C. M. Mulvenna** is a Psychologist at the School of Psychology, University of Glasgow, UK; **C. Maclean** is a Clinical Psychologist at the Royal Edinburgh Hospital, UK; and **A. Polnay** is a Consultant Psychiatrist in Psychotherapy at State Hospital, Carstairs, and an Honorary Fellow at the Division of Psychiatry, University of Edinburgh, UK

## Appendix 1

### Mentalising Skills Training Questionnaire

**Thank you for your time. Please answer all of the questions.**

**Have you previously attended a course in Mentalising?**

**Table:**

YES			NO		

	Strongly Disagree	Disagree	Neutral	Agree	Strongly Agree
1. I believe mentalising is simple to put into practice					
2. I think mentalising skills have a theoretical basis					
3. I think mentalising skills help promote therapeutic communication with people who have problems like Borderline and Anti-Social Personality Disorder					
4. I think providing mentalising skills requires a specialist psychotherapist					
5. I believe treating people using psychological techniques is a poor use of resources					
6. I think childhood experiences can have a profound impact on adult relationships					
7. Mentalisation therapies do not allow patients to take medication					
8. A person saying that a visit in the middle of the night from her partner was the only way she was reassured he loved her is an example of a teleological stance					
9. Breaks in therapy (or from relationships with people) are not considered important by MBT					
10. A key component of mentalising is thinking about people's attachment relationships					
11. A therapist using mentalising skills will be expressionless					
12. Using mentalising, you can ask questions to promote exploration					
13. Mentalising avoids exploring the current therapeutic relationship with the person you are working with					
14. In mentalising, professionals do not consider their own feelings					
15. The professionals might use a ‘stop and stand’ technique if they get muddled in mentalising					
16. As mentalising is straightforward, there is no need for supervision					
17. Strong disagreements between professionals about a person's management may be an indication of non-mentalising					
18. A partner being certain about what people are thinking, without checking, is an example of psychic equivalence					
19. I feel confident in applying MBT skills into working practice					
20. I understand when to apply/use MBT skills					

## Appendix 2

### Attitudes to Personality Disorder Questionnaire

For the purposes of this questionnaire we would like you to think about your feelings towards patients with personality disorder (PD) overall. We realise that you may have different mixtures of feelings about different personality disordered patients you have cared for in the past. For this questionnaire we would like to you try and average those out and tell us what your responses are in general towards patients with personality disorder as a whole.

For each response listed below please indicate the frequency of your feelings towards people with a personality disorder. Please circle your choice quickly, rather than spending a long time considering it. We want to know your honest, gut feelings.

**Table:**

		Never	Seldom	Occasionally	Often	Very often	Always
1	I like PD people	1	2	3	4	5	6
2	I feel frustrated with PD people	1	2	3	4	5	6
3	I feel drained by PD people	1	2	3	4	5	6
4	I respect PD people	1	2	3	4	5	6
5	I feel fondness and affection for PD people	1	2	3	4	5	6
6	I feel vulnerable in PD people company	1	2	3	4	5	6
7	I have a feeling of closeness with PD people	1	2	3	4	5	6
8	I feel manipulated or used by PD people	1	2	3	4	5	6
9	I feel uncomfortable or uneasy with PD people	1	2	3	4	5	6
10	I feel I am wasting my time with PD people	1	2	3	4	5	6
11	I am excited to work with PD people	1	2	3	4	5	6
12	I feel pessimistic about PD people	1	2	3	4	5	6
13	I feel resigned about PD people	1	2	3	4	5	6
14	I admire PD people	1	2	3	4	5	6
15	I feel helpless in relation to PD people	1	2	3	4	5	6
16	I feel frightened of PD people	1	2	3	4	5	6
17	I feel angry towards PD people	1	2	3	4	5	6
18	I feel provoked by PD people behaviour	1	2	3	4	5	6
19	I enjoy spending time with PD people	1	2	3	4	5	6
20	Interacting with PD people makes me shudder	1	2	3	4	5	6
21	PD people make me feel irritated	1	2	3	4	5	6
22	I feel warm and caring towards PD people	1	2	3	4	5	6
23	I feel protective towards PD people	1	2	3	4	5	6
24	I feel oppressed or dominated by PD people	1	2	3	4	5	6
25	I feel that PD people are alien, other, strange	1	2	3	4	5	6
26	I feel understanding towards PD people	1	2	3	4	5	6
27	I feel powerless in the presence of PD people	1	2	3	4	5	6
28	I feel happy and content in PD people company	1	2	3	4	5	6
29	I feel cautious and careful in the presence of PD people	1	2	3	4	5	6
30	I feel outmanoeuvered by PD people	1	2	3	4	5	6
31	Caring for PD people makes me feel satisfied and fulfilled	1	2	3	4	5	6
32	I feel exploited by PD people	1	2	3	4	5	6
33	I feel patient when caring for PD people	1	2	3	4	5	6
34	I feel able to help PD people	1	2	3	4	5	6
35	I feel interested in PD people	1	2	3	4	5	6
36	I feel unable to gain control of the situation with PD people	1	2	3	4	5	6
37	I feel intolerant. I have difficulty tolerating PD people behaviour	1	2	3	4	5	6
